# SGLT2-is in Acute Heart Failure

**DOI:** 10.3390/jcm14217799

**Published:** 2025-11-03

**Authors:** Matteo Bianco, Concetta Di Nora, Renata De Maria, Amir Hassan Mousavi, Samuela Carigi, Luisa De Gennaro, Paolo Manca, Maria Vittoria Matassini, Vittoria Rizzello, Maria Denitza Tinti, Giovanna Geraci, Attilio Iacovoni, Furio Colivicchi, Massimo Grimaldi, Fabrizio Oliva

**Affiliations:** 1 Area Scompenso Cardiaco, Associazione Nazionale Medici Cardiologi Ospedalieri (ANMCO), 50121 Firenze, Italy; concetta.dinora@gmail.com (C.D.N.); samuela.carigi@auslromagna.it (S.C.);; 2 Division of Cardiology, San Luigi Gonzaga University Hospital, 10043 Orbassano, Italy; amir.mousavi@edu.unito.it; 3 Department of Cardiothoracic Science, Azienda Sanitaria Universitaria Friuli Centrale, 33100 Udine, Italy; 4 Department of Primary Care, ASST Grande Ospedale Metropolitano Niguarda, 20121 Milano, Italy; 5 Cardiology Unit, Infermi Hospital, Azienda Unità Sanitaria Locale della Romagna, 47923 Rimini, Italy; 6 Cardiology Department, San Paolo Hospital, 70123 Bari, Italy; 7 Department of Clinical Cardiology and Heart Failure, Mediterranean Institute for Transplantation and Advanced Specialized Therapies, ISMETT IRCCS, 90121 Palermo, Italy; 8 Cardiac Intensive Care Unit-Cardiology Division, Department of Cardiovascular Sciences, Azienda Ospedaliera Universitaria delle Marche, 60124 Ancona, Italy; 9 Unit of Cardiology, Azienda Ospedaliera San Giovanni Addolorata, 00184 Roma, Italy; 10 Unit of Cardiology, Azienda Ospedaliera San Camillo Forlanini, 00152 Roma, Italy; 11 Unit of Cardiology and Intensive Care Unit, Ospedale Sant’Antonio Abate, 91016 Trapani, Italy; 12 SSD Chirurgia dei Trapianti e del Trattamento Chirurgico dello Scompenso, Dipartimento Cardiovascolare, ASST Papa Giovanni XXIII, 24127 Bergamo, Italy; 13 Clinical and Rehabilitation Cardiology Division, San Filippo Neri Hospital—ASL Roma 1, 00135 Roma, Italy; 14 Unit of Cardiology and Intensive Care Unit, Miulli Hospital, Acquaviva delle Fonti, 70021 Bari, Italy; 15 Cardiologia 1-Emodinamica, Dipartimento Cardiotoracovascolare “A. De Gasperis”, ASST Grande Ospedale Metropolitano Niguarda, 20162 Milano, Italy

**Keywords:** SGLT2i, acute heart failure, decongestion

## Abstract

Despite the wealth of evidence in favour of SGLT2 inhibitor use in patients with chronic heart failure, their role in the very early stages of heart failure is still unclear. While the latest update of the European Society of Cardiology guidelines on heart failure advocates the use SGLT2 inhibitors in the acute phases of heart failure based on the results of the latest trials, it does not clarify the appropriate timing to start this therapy, leaving the clinician to decide whether SGLT2 inhibitors should be started directly during hospitalization or at discharge. Conversely, the recently published focused update of the American College of Cardiology expert consensus decision pathway on the clinical assessment, management, and trajectory of patients hospitalized with heart failure clearly supports the safety and early clinical benefit use of SGLT2 inhibitors based on evidence coming from the EMPULSE and SOLOIST-WHF trials. The expert consensus decision pathway states that SGLT2 inhibitors can be initiated regardless of left ventricular ejection fraction at any time during hospitalization and places a greater emphasis on implementing the other pillars of therapy for heart failure with reduced ejection fraction after stabilization. Moreover, the results of the very recent DAPA ACT HF–TIMI 68 trial on dapagliflozin in patients with acute heart failure, although limited by a follow-up of only 2 months, did not show a reduction in mortality or heart failure hospitalizations. Based on the currently available published data, we will review what is already known about the use of these drugs in the early phases of acute heart failure and analyze their pathophysiological rationale from a practical perspective.

## 1. Upfront Utilization of SGLT2 Inhibitors in Acute Heart Failure

After the publication of the recent heart failure (HF) guidelines [1], SGLT2 inhibitors (SGLT2i) were recognized as one of the recommended treatments in patients with HF and reduced ejection fraction (HFrEF) irrespective of the presence of type 2 diabetes mellitus (class I recommendation, level of evidence A) due to the demonstrated reduction in mortality and hospitalization rates in these patients.

Shortly afterwards, important clinical evidence in favour of SGLT2i use in patients with HF and preserved ejection fraction (HFpEF) became available and it was taken up by the subsequently published [2,3] American and European guidelines. SGLT2is have indeed been shown to reduce cardiovascular mortality and hospitalizations for HF decompensation, even in the population of patients with HFpEF (recommendation class IIa, level of evidence B).

At variance with the strong evidence in the chronic HF setting, the management of the acute patient still leaves room for some doubt. Endocrinology and diabetes guidelines advise against SGLT2i use during hospitalizations for acute episodes due to the potential increase in adverse events such as genitourinary infections, euglycemic ketoacidosis, and acute renal failure [4,5,6]. The 2023 update of the European Society of Cardiology Heart Failure Guidelines introduced the possibility of using SGLT2is in acute HF based on the results of the EMPULSE study, but it did not provide any recommendations regarding the best time to start therapy (during hospitalization vs. at discharge) [7]. Conversely, the recently published update on the decision-making and management pathway for patients hospitalized for HF by the American College of Cardiology (ACC) clearly states that SGLT2i therapy can be started any time during HF hospitalization, regardless of ejection fraction (EF), while placing more emphasis on the initiation of the other pillars of HFrEF therapy only after stabilization [8].

**Figure 1:**
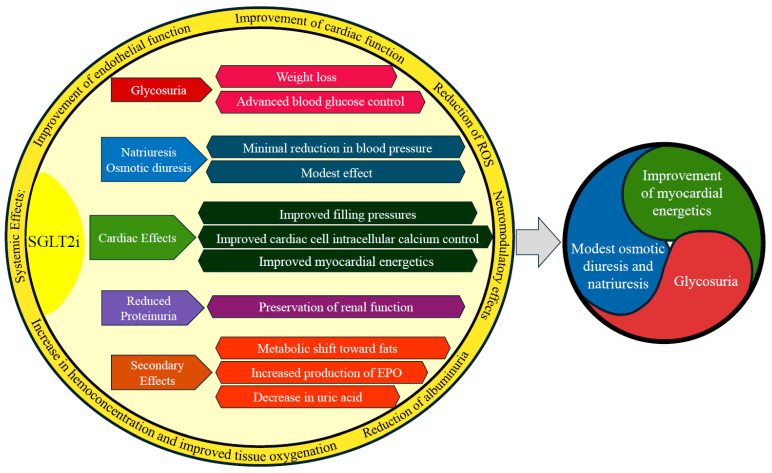
Summary of the main effects of SGLT2is. Abbreviations: ROS, reactive oxygen species; EPO, erythropoietin.

For many years, drugs that have been tested for efficacy in the setting of acute HF have produced disappointing results, and the therapy of this condition is still largely based on the clinical experience of the attending cardiologist. The strong evidence of the efficacy and safety of the ‘four pillars’ of HFrEF therapy demonstrated in the registration trials has prompted the quest for the optimal timing and strategy to initiate such therapies. While expeditious introduction near hospital discharge and the rapid titration of drugs in the first month post-discharge were shown to reduce mortality and HF hospitalizations [9,10], it is less clear which drugs should be administered early during the decongestion phase of hospitalization. Beyond their long-term prognostic benefit, SGLT2is exhibit some peculiarities that might favour their use early during an acute HF admission, which include the following: 1. a potential synergistic effect with loop diuretics in achieving decongestion; 2. ease of use and a high safety and tolerability profile, with negligible effects on arterial blood pressure and no effect on heart rate; 3. the ability to counterbalance the risk of hyperkalemia associated with starting drugs that act on the renin–angiotensin–aldosterone axis (RAAS); and 4. the improvement in cardiomyocyte metabolism in a phase of high oxidative stress such as acute HF (Figure 1).

In this review, we will examine the available data for the use of SGLT2is in the acute phase of HF, critically discuss the pathophysiological rationale for their use, and attempt to provide practical guidance.

## 2. Early Benefit from Trials on SGLT2is in Chronic HF

The data in favour of the use of SGLT2is in patients with chronic HF are robust. Both empaglifozin, in the EMPEROR-Reduced [2] and EMPEROR-Preserved [3] trials, and dapaglifozin, in the DAPA-HF [11] and DELIVER [12] trials, demonstrated significant reductions in the primary endpoint of cardiovascular mortality and HF hospitalization with respect to placebo across the range of reduced and preserved EF. Of particular relevance to their potential use in the acute HF stage was the documentation of early benefit after drug initiation. In DAPA-HF [11], the reduction in the primary outcome was already evident after 28 days of therapy (hazard ratio, 0.51; 95% confidence interval [CI], 0.28 to 0.94). In EMPEROR-Reduced [2], a statistically significant reduction in cardiovascular mortality emerged between the two arms (hazard ratio [HR] 0.42; 95% CI 0.19–0.92) after only 12 days of treatment start. The DELIVER [12] trial also enrolled inpatients hospitalized for acute HF (10.4%) and documented a reduction in the primary endpoint (HR 0.82, 95% CI 0.73–0.92; *p* < 0.001) in patients randomized to dapaglifozin vs. placebo both during a HF hospitalization and in a stable clinical state [13], without differences according to EF level or the presence or absence of diabetes. These large and early benefits, documented even in patients with a recent HF admission, have paved the way to an early use of SGLT2is in the acute HF stages.

## 3. Studies on the Use of SGLT2is in Acute Heart Failure

Several studies investigated the impact of SGLT2is during a hospitalization for acute HF or in the first 30 days after discharge (Table 1).

## 4. Empagliflozin

EMPAG-HF was the first randomized double-blind study to test the use of empagliflozin 25 mg or placebo in addition to standard decongestant therapy during the first 5 days of hospitalization for acute HF in 60 patients. The work showed a significant increase in total diuresis (10.8 litres vs. 8.7 litres; mean difference 2.2 litres; 95% CI 0.84–3.6; *p* = 0.003) and a better efficiency of the loop diuretic in terms of millilitres of diuresis/milligram of furosemide administered (8.3 mL/mg vs. −25.9 mL/mq; mean difference 43.7 mL/mg; 95% CI 0.1–0.93; *p* = 0.041) after 5 days of treatment compared to the placebo group [22].

The subsequent EMPA-RESPONSE-AHF trial randomized 80 patients, with and without diabetes, with clinical signs of congestion, need for intravenous diuretic therapy, and an NT-ProBNP ≥ 1400 pg/mL [15] to treatment with empagliflozin 10 mg or placebo within 24 h of admission for acute HF. No significant differences were documented in the primary endpoint, improvement in dyspnea according to a predetermined score, response to diuretic therapy, duration of hospitalization, and reduction in NT-ProBNP levels. However, the drug was well tolerated, and an increase in urinary volume and a reduction in HF episodes, re-hospitalizations for HF decompensation, and cardiovascular death at 60 days were observed. The results of this pilot trial formed the rationale for the recently published [16] double-blind, randomized EMPULSE study. This trial randomized 530 patients with newly diagnosed or relapsed HF to empagliflozin 10 mg or placebo. Of the 530 randomized patients, 361 had HFrEF and 169 HfpEF. The median time to randomisation was 3 days (range 2–4 days) since admission to hospital for HF. The primary endpoint was death from all causes, first HF hospitalization, total number of HF hospitalizations, and change from baseline of at least five points on the Kansas City Cardiomyopathy Questionnaire (KCCQ-TSS). The safety parameters taken into account were volume depletion, hypotension, and renal failure. Empagliflozin was superior to placebo in 53.9% of paired comparisons and placebo superior to empagliflozin in 39.7%. Empagliflozin benefit was independent of both HF type (de novo vs. recurrent) and EF (≤40% vs. >40%). The incidence of adverse events was overall lower in patients treated with empagliflozin compared to placebo, while the drug was discontinued due to adverse events in 8.5% and 13%, respectively.

## 5. Sotagliflozin

The SOLOIST-WHF study is a randomized double-blind trial that tested the use of sotagliflozin (SLGT2-SGLT1 inhibitor) vs. placebo in patients with type 2 diabetes mellitus hospitalized for acute HF [23]. The trial enrolled 1222 patients and 79% of these had an EF < 50%, so both patients with HFrEF and HFpEF were included. Treatment with sotagliflozin could be started as early as on the first day of hospitalization and no later than three days after discharge, after switching from intravenous to oral diuretics, in all hemodynamically stable patients with a systolic blood pressure above 100 mmHg. The trial was stopped early due to discontinuation of financial support by the sponsor but still demonstrated a reduction in the primary outcome, the composite of death from cardiovascular causes and emergency admissions/visits for HF, after a mean follow-up of 12 months (HR 0.67; 95% CI 0.52–0.85; *p* < 0.001). The primary outcome was predominantly determined by a reduction in emergency admissions/visits for HF (HR 0.64; 95% CI 0.49–0.83; *p* < 0.001). A higher incidence of episodes of diarrhea (6.9% vs. 4.1%, *p* = 0.032) and severe hypoglycemia (1.5% vs. 0.3%, *p* = 0.037) occurred in the sotagliflozin group. These latter adverse effects appear to be related to SGLT1 inhibition by sotagliflozin. A subsequent sub-analysis in patients starting sotagliflozin during an HF hospitalization also showed a reduction in total mortality at 30 and 90 days [14].

## 6. Dapagliflozin

The efficacy and safety of dapagliflozin on top of standard decongestion therapy in patients with de novo or exacerbated acute HF was initially tested in several small randomized trials. The primary endpoints of these studies were related to decongestion measures such as weight loss, improvement in dyspnea, reduction in NT-ProBNP levels, and efficiency of loop diuretics.

In the study by Ibrahim et al. [20], 100 patients with type 2 diabetes mellitus, LVEF≤ 40%, and a history of HF with an ongoing exacerbation (defined according to clinical criteria) were randomized to placebo or dapagliflozin 10 mg on top of conventional decongestion. The study showed an increase in urinary output in the dapagliflozin-treated group (18.46 ± 0.5 litres vs. 14.43 ± 0.7 litres; *p* < 0.001), better efficiency of the loop diuretic in terms of ml of diuresis/mg of furosemide administered (34.75 ± 2.2 mL/mg vs. 19.49 ± 1.2 mL/mg; *p* < 0.001), and a greater weight loss (76.51 ± 6.0 kg vs. 79.63 ± 8.9 kg, *p* = 0.046) against a non-statistically significant trend of worsening renal function in the dapagliflozin-arm (28 vs. 16%, *p* = 0.148). These results were more recently confirmed by the DAPA-RESPONSE-AHF and the DICTATE-AHF trials [17,19]. The DAPA-RESPONSE-AHF is a similarly designed double-blind trial that also included patients without type 2 diabetes mellitus and showed not only a reduction in congestion by adding dapagliflozin to standard therapy but also a reduction in re-hospitalizations at 30 days (*p* = 0.017). DICTATE-AHF tested the use of dapagliflozin in combination with a standardized titration of loop diuretic therapy in order to reach a daily diuresis between 3 and 5 litres. Unlike previous studies, which allowed liberal use of diuretic therapy, DICTATE-AHF standardized the use of the loop diuretic and excluded patients treated with doses ≥ 12.5 mg hydrochlorothiazide in order to avoid potential bias. No significant differences emerged with regard to the primary outcome of the study, which was the efficiency of diuretic therapy measured as a reduction in body weight in relation to the cumulative dose of loop diuretic after 5 days of therapy ((−0.42; interquartile range −0.52 to −0.33) Kg/40 mg furosemide vs. −0.31 (interquartile range −0.39 to −0.23) Kg/40 mg furosemide; mean difference 0.65: 95% CI 0.41–1.02; *p* = 0.06)). However, implementation in the first 24 h of dapagliflozin was safe. There was no increase in hypoglycemic episodes nor a significant worsening glomerular filtration rate (−2 mL vs. −3.2 mL; mean difference 1.06 mL: 95% CI 0.68–1.65; *p* = 0.79) and no episodes of euglycemic ketoacidosis were observed. Dapagliflozin was also compared with metolazone in patients with resistance to loop diuretics in the DAPA-RESIST study [18]. In this multicentre randomized study, 61 patients hospitalized for an acute HF exacerbation were randomized in the first 24 h of hospitalization to therapy with dapagliflozin 10 mg or metolazone 5–10 mg on top of standard decongestion for the first 3 days of hospitalization. The study did not show a statistically significant difference in the primary endpoint, weight loss at 96 h after hospitalization (−3 kg vs. −3.6 kg; mean difference 0.65 kg; 95% CI −0.12–1.41; *p* = 0.11), nor in the efficiency of diuretic therapy (−0.15 kg/40 mg furosemide vs. −0.25 kg/40 mg furosemide; mean difference −0.08: 95% CI −0.17–0.01; *p* = 0.11), but the increase in urea and creatinine was lower with dapagliflozin than with metolazone.

Recently, a larger randomized trial has tested dapagliflozin in the setting of acute HF. The DAPA ACT HF-TIMI 68 study, a randomized double-blind study, enrolled 2401 patients with acute HF to assess whether initiating dapagliflozin in the acute phase is able to reduce cardiovascular mortality or worsening HF, defined as deterioration during the index hospitalization, re-hospitalization, or urgent visit for HF in the first 2 months after discharge. Enrolment was independent of LVEF, presence of type 2 diabetes, and de novo or worsening chronic HF. Participants were randomized to blinded treatment for a 2-month period. Key safety endpoints comprise symptomatic hypotension and decline in renal function. The composite endpoint occurred in 10.9% of patients receiving dapagliflozin and 12.7% of those on placebo, with no statistically significant difference (HR 0.86; 95% CI 0.68–1.08; *p* = 0.20). All individual components of the composite primary endpoint were numerically lower with dapagliflozin but none reached statistical significance: cardiovascular mortality (2.5% vs. 3.1%; HR 0.78; 95% CI 0.48–1.27), all-cause mortality (3.0% vs. 4.5%; HR 0.66; 95% CI 0.43–1.00), and HF worsening (7.4% vs. 8.6%; HR 0.85; 95% CI 0.64–1.13). Finally, the safety endpoints were reassuring, with only modestly higher rates of symptomatic hypotension (3.6% vs. 2.2%) and numerically higher renal function decline (5.9% vs. 4.7%) among patients treated with dapagliflozin [21]. These data suggest that in more vulnerable populations, care should be taken to minimize other potential drivers of symptomatic hypotension, such as volume depletion or rapid escalation in the dosing of neurohormonal antagonists, when initiating SGLT2is.

All these data, together with the results of some meta-analyses [24,25], support the implementation of SGLT2is already during the early stages of HF hospitalization. As explicitly stated in the ACC document [8], SGLT2is and mineralocorticoid antagonists have little effect in reducing arterial blood pressure and, in the absence of contraindications, can be started at any time during hospitalization and continued after discharge. This would allow a subsequent rapid implementation of the other guideline-recommended treatment for HF. It is important to emphasize that in clinical practice, the implementation of such therapy is often not so rapid due to the fear that the combined effect of all the recommended drugs may not be tolerated. In particular, early and concomitant initiation of ARNI and SGLT2is could affect arterial blood pressure and renal function, which are a well-known and critical issue in these patients. Of note, neuromodulation through inhibition of the RAAS, neprilysin, and beta-adrenergic systems has potent effects on the circulation and requires careful titration compared to SGLT2 and MRA inhibitors, which have a trivial effect on reducing arterial blood pressure and do not impact cardiac output. Therefore, unlike SGLT2is, the introduction and optimization of neurohormonal modulation will depend mainly on the hospital course and the patient’s characteristics/profile during the hospital stay [26].

## 7. Mechanisms of SGLT2is in Acute Heart Failure

**Figure 2:**
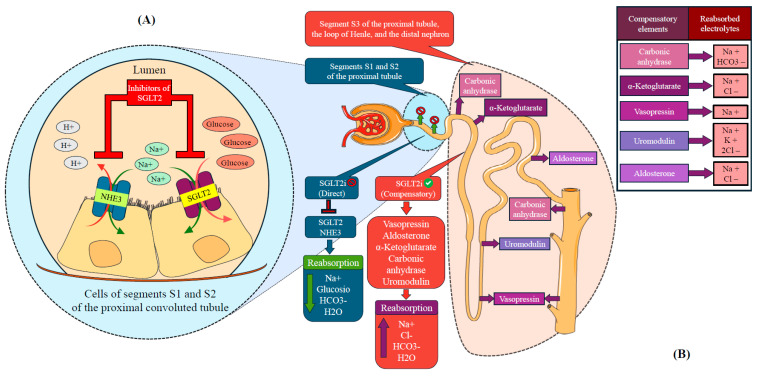
Site of action and main compensatory mechanism of SGLT2is. (**A**): Site of action of SGLT2is in the kidney. (**B**): Main compensatory mechanism of SGLT2is in patients with acute heart failure.

SGLT2is act primarily at the proximal tubule level, where they block sodium–glucose reabsorption via the SGLT2 co-transporter, thereby promoting osmotic diuresis. During acute decompensation, sympathetic hyperactivity upregulates renal SGLT2 expression, amplifying glycosuria in this setting [27]. In addition, SGLT2 activity modulates the sodium–hydrogen (NH3) exchanger at the proximal tubule; its inhibition contributes to reduced sodium reabsorption [28] (Figure 2A). However, SGLT2 and NH3 inhibition is rapidly counterbalanced by compensatory mechanisms at the loop of Henle and distal tubule, triggered by elevated sodium chloride delivery. These include increased aldosterone and vasopressin release, enhanced sodium and bicarbonate reabsorption through carbonic anhydrase, and vasoconstriction of the afferent arteriole reducing glomerular filtration. As a result, SGLT2is induce only a short-lived osmotic diuresis, followed by strong activation of water- and sodium-retaining mechanisms, particularly in patients with acute HF (Figure 2B). Thus, their direct decongestive effect appears physiologically modest [29].

Studies assessing plasma volume have reported only modest reductions, which are later offset by increased red blood cell mass due to SGLT2i-induced erythropoiesis [30]. This mechanism also explains the limited effect on natriuretic peptide levels, which decline only slightly in acute HF [31]. Early studies suggested that SGLT2is may reduce extracellular volume more effectively than loop diuretics [32]. However, the decongestive action of loop diuretics results from plasma volume reduction, which promotes capillary refill from the interstitial space and increased lymphatic flow, ultimately leading to fluid excretion and edema resolution [33]. The hypothesis that SGLT2is sustain aquaresis and plasma osmolarity, thereby mobilizing edema, does not account for their erythropoiesis-driven plasma volume expansion and has not been confirmed by clinical data [28].

A more plausible pathophysiological mechanism for SGLT2is’ role in decongestion may lie in offsetting loop diuretic resistance. Resistance is driven by hypochloremia, which enhances sodium–potassium–chloride co-transporter activity at the loop of Henle, the target of loop diuretics. By raising chloride concentration at this site, SGLT2is may attenuate resistance, thereby augmenting loop diuretic efficacy. This effect is likely more significant in chronic HF, consistent with conflicting data on loop diuretic efficiency in acute settings with SGLT2i. Nonetheless, their efficacy, as shown in the DAPA-RESIST trial, remains inferior to the effect of thiazides within sequential nephron blockade, albeit with greater renal safety [18,34].

The EMPULSE trial, the largest to date evaluating empagliflozin in acute HF, reported significant benefits in a post hoc analysis: greater weight loss (−3.2 vs. −1.23 kg; mean difference −1.97 kg; 95% CI −2.86 to −1.08; *p* < 0.0001), improved loop diuretic efficiency (−3.33 vs. −1.02 kg/40 mg furosemide; mean difference −2.31 kg; 95% CI −3.77 to −0.85; *p* = 0.0020), reduced NT-proBNP (ratio of adjusted geometric mean 0.92; 95% CI 0.86–0.98; *p* = 0.0101), and improvement in congestion score (−1.78 vs. −1.43 points; mean difference −0.34; 95% CI −0.60 to −0.09; *p* = 0.0079) at 15 days [31]. However, urinary sodium or volume data were not presented. Thus, it remains uncertain whether these results reflect direct decongestion (free water/sodium excretion) or rather weight loss driven by glycosuria and concomitant caloric restriction in acutely decompensated patients [35]. Overall, the evidence suggests a modest decongestive role for SGLT2i.

**Figure 3:**
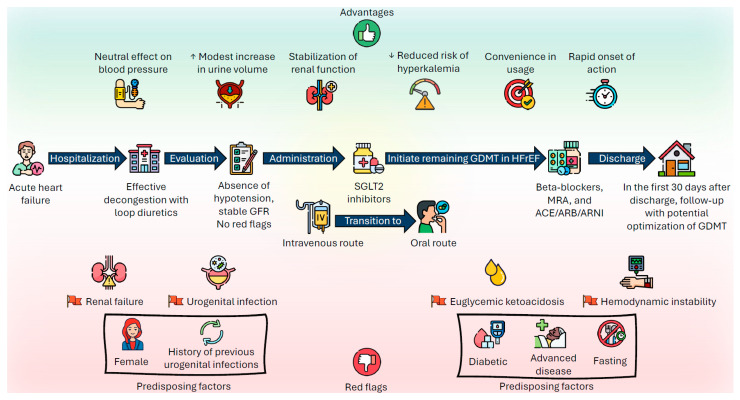
Practical approach to the use of SGLT2is in patients with acute decompensated heart failure.

Importantly, the early clinical benefits of SGLT2i in acute HF may be mediated by direct effects on the cardiomyocyte. By mimicking fasting, they enhance fatty acid and ketone body metabolism, reduce oxidative stress, promote autophagy, and ultimately protect cardiomyocytes. In acute HF, these mechanisms may translate into reduced re-hospitalization and mortality as early as 30 days after therapy initiation [25]. For this reason, even if their decongestive capacity is limited, early initiation during hospitalization is essential. In a patient is already on SGLT2i therapy, continuation during decompensation appears reasonable—provided that the patient is hemodynamically stable and has no clear contraindications such as severe renal deterioration (Figure 3) [36,37]. In Table 2, we have summarized the advantages and disadvantages of SGLT2i therapy usage.

## 8. Red Flags for Deferred Use

Safety is among the features that have emerged most robustly from the clinical trials that have tested SGLT2is in all their fields of application. However, there are clinical scenarios in which initiation should be postponed in order to avoid adverse effects that, although rare, can sometimes be serious:

1. Hemodynamic instability: SGLT2 inhibitors have not been tested in hemodynamically unstable patients and therefore these drugs should not be started in patients requiring inotropes, vasopressors, intravenous vasodilators, or short-term mechanical circulatory support. It appears advisable to start SGLT2is when systolic blood pressure is above 95 mmHg, in the absence of hypotensive episodes for at least 12 h [24,28].

2. Renal insufficiency: Dapagliflozin and empagliflozin have been shown to be safe down to a glomerular filtration rate of 25 and 20 mL/min, respectively, and to slow the progressive deterioration of renal function in chronic patients. In the acute phase, and particularly during intravenous diuretic therapy, the risk of volume depletion associated with loop diuretics and/or sequential nephron blockade may cause acute renal failure. Starting SGLT2is during an acute decompensation hospitalization should therefore be postponed in case of excessive fluid depletion. Monitoring of fluid status and renal function during hospitalization is crucial, particularly in the case of concomitant initiation of sacubitril/valsartan [24].

3. Genitourinary tract infections: Diabetic patients, women, and patients with obesity had a higher incidence of urinary tract infections in trials. Such events were infrequent in studies testing SGLT2is in the acute phase. It is recommended to postpone SGLT2i initiation in case of ongoing genitourinary tract infections, particularly in subjects with recurrent episodes, patients with diabetes, or with other predisposing factors for infections [24].

4. Euglycemic ketoacidosis: This is an extremely rare but potentially fatal complication, particularly in patients with acute HF. In this phase, the coexistence of prolonged fasting and hypotension can cause an insulin deficit that favours the onset of ketoacidosis. This condition is almost exclusive to insulin-treated diabetics and occurred in 2 patients out of the approximately 900 included in the SOLOIST-WHF, EMPULSE, and EMPA-RESPONSE-AHF studies. Awareness of its possible occurrence is crucial in order to institute prompt treatment. To prevent the risk of euglycemic ketoacidosis, SGLT2is should be started after resumption of oral feeding in patients admitted for acute HF decompensation [24].

5. The data available until now on heart and kidney transplant recipients treated with SGLT2is seems to be promising in this subgroup of patients as well; however, the risk of genitourinary infections could be higher in this population due to their chronic immunosuppressant drug regimen.

**Figure 4:**
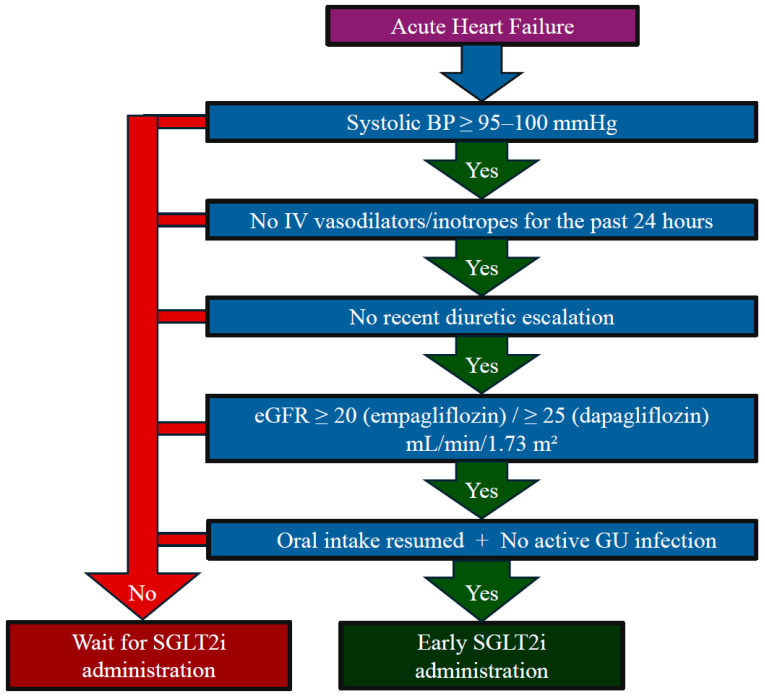
Flowchart for practical use of SGLT2is in patients with acute heart failure.

In Figure 4, we have summarized a practical flowchart for initiating SGLT2 inhibitors in patients with acute HF.

## 9. Conclusions

Acute HF is a frequent cause of emergency room admission and hospitalization, associated with high in-hospital mortality and short-term re-hospitalization. Therapeutic optimization and early treatment are the key to reducing these adverse outcomes. The use of SGLT2is in patients with acute HF is reassuring, and their use appears to be safe. Moreover, although studies show a rather modest direct decongestive effect, early introduction should always be pursued, in the absence of contraindications, as it brings prognostic advantages and facilitates the introduction of the other guideline-recommended treatments for heart failure.

## Figures and Tables

**Table 1:** Summary of the main studies testing the use of SGLT2 inhibitors in patients with acute heart failure.

Study	Type of SGLT Inhibition	Comparison Groups	Main Eligibility Criteria	Method of Initiating SGLTis	Follow-Up	Primary Outcomes	Overall Treatment Effect	Relevant Data
SOLOIST-WHF [14]	SGLT1 and SGLT2	Sotagliflozin 200 mg once daily (titrated up to 400 mg) vs. placebo (*n* = 1222)	Reduced and preserved LVEF Type 2 diabetes eGFR ≥ 30 mL/min/m^2^	Before discharge (48.8%) Right after discharge (median 2 days, 51.2%)	9 months	Total number of CV deaths and hospitalizations and urgent visits for heart failure	51.0 vs. 76.3 events per 100 patient-years Hazard ratio 0.67 (95% CI 0.52–0.85)	Early termination of the trial due to loss of funding by the sponsor Benefit driven by a reduction in hospitalizations and visits for heart failure Benefit consistent across subgroups and treatment initiation methods Higher incidence of diarrhea and severe hypoglycemia in the sotagliflozin group
EMPA-RESPONSE-AHF [15]	SGLT2	Empagliflozin 10 mg once daily vs. placebo for 30 days (*n* = 80)	Clinical signs of congestion NT-proBNP ≥1400 pg/mL Need for initiation of loop diuretics	Within 24 h of hospitalization	60 days	Change in dyspnea VAS score, NT-proBNP, diuretic response, and length of hospital stay	Mean combined difference in various secondary endpoints −0.019 (95% CI −0.306–0.269)	No significant difference in any primary outcomes Reduction in a combined secondary endpoint of in-hospital HF worsening, HF hospitalization, or death at 60 days vs. placebo Increased urine output by day 4 Safety and tolerability. No adverse effects on blood pressure or renal function
EMPULSE [16]	SGLT2	Empagliflozin 10 mg once daily vs. placebo (*n* = 530)	NT-proBNP ≥1600 pg/mL Furosemide dose ≥40 mg IV eGFR ≥ 20 mL/min/m^2^	From the first to the fifth day after hospitalization	90 days	Combination of death, number of HF episodes, time to first HF event, and change in KCCQ-TSS	Clinical benefit 53.9% vs. 39.7% Win ratio 1.36 (95% CI 1.09–1.68)	Significant numerical reduction in deaths and HF events with empagliflozin Benefit also demonstrated with standard survival analysis Early and sustained weight loss of 1.5 kg Serious adverse events were more frequent in the placebo group No cases of ketoacidosis
DAPA-RESPONSE-AHF [17]	SGLT2	Dapagliflozin 10 mg once daily vs. placebo (*n* = 87)	Age ≥ 18 Acute HF with dyspnea at rest or minimal exertion At least one additional sign of congestion History of type 2 diabetes Newly diagnosed HbA1c ≥ 6.5% at hospitalization	Within 24 h of hospitalization up to 30 days	From day 4 to 60 days post-hospitalization (30 days post-treatment)	Difference between groups in AUC of VAS dyspnea score during the first 4 days	Dapagliflozin significantly reduced the AUC of VAS dyspnea score vs. placebo (3192.2 ± 1631.9 mm × h vs. 4713.1 ± 1714.9 mm × h, *p* < 0.001)	Greater relative NT-proBNP reduction from baseline with dapagliflozin (−34.89% vs. −10.085%, *p* = 0.001) Higher cumulative urine volume after 4 days of therapy (18,600 mL with dapagliflozin vs. 13,700 mL with placebo, *p* = 0.031) Dapagliflozin reduced the number of re-hospitalizations within 30 days of discharge Dapagliflozin did not affect urinary Na+ concentration, HF worsening incidence, or mortality
DAPA-RESIST [18]	SGLT2	Dapagliflozin 10 mg once daily vs. metolazone 5–10 mg once daily for 3 days (*n* = 61)	Patients hospitalized for HF Resistance to IV furosemide therapy BNP ≥ 100 pg/mL or NT-proBNP ≥ 400 pg/mL Persistent congestion Expected hospital stay >3 days	For up to three consecutive days after admission	5 days	Diuretic effect assessed by weight change (kg), pulmonary congestion changes (lung ultrasound), loop diuretic efficiency (weight change per 40 mg furosemide), and congestion assessment score	Mean weight reduction at 96 h: 3.0 kg with dapagliflozin vs. 3.6 kg with metolazone [mean difference 0.65, 95% CI −0.12, 1.41 kg; *p* = 0.11]	Loop diuretic efficiency was lower with dapagliflozin vs. metolazone Pulmonary congestion changes were similar between treatments Plasma sodium and potassium decreases and urea and creatinine increases were smaller with dapagliflozin than with metolazone Serious adverse events were similar between treatments Dapagliflozin patients received more furosemide but had fewer biochemical alterations than metolazone patients Dapagliflozin was not more effective than metolazone in alleviating congestion
DICTATE-AHF [19]	SGLT2	Dapagliflozin 10 mg once daily vs. protocolized diuretic titration until day 5 or hospital discharge (*n* = 240)	Adults with type 2 diabetes eGFR ≥ 30 mL/min/1.73 m^2^ Hospitalized with AHF Planned or ongoing IV loop diuretics Hypervolemia and randomization within 24 h of ER presentation or direct admission to hospital	For up to five consecutive days of admission or until hospital discharge	30 days post-discharge (telephone follow-up for outcome assessment)	Concentration of natriuretic peptides, weight, and congestion assessment using edema scale at day 5 or discharge	Dapagliflozin is not associated with greater body weight reduction after 5 days of treatment (−0.42 kg/40 mg furosemide vs. −0.31 kg/40 mg furosemide, CI 0.41–1.02; *p* = 0.06)	No difference was found between the diuretic efficacy of dapagliflozin and that of usual therapy Dapagliflozin was associated with reduced loop diuretic doses Early dapagliflozin initiation did not increase diabetic, renal, or cardiovascular adverse events. Dapagliflozin improved mean 24 h natriuresis and urine production, accelerating hospital discharge
Ibrahim et al. 2020 [20]	SGLT2	Dapagliflozin 10 mg once daily alone or with insulin (as needed) and furosemide (*n* = 100)	Age > 18, Type 2 diabetic patients with HF history Indication for AHF hospitalization ≥1 symptom: respiratory distress or orthopnea ≥1 clinical sign of congestion (peripheral edema, jugular venous distension, or pulmonary congestion signs) Chronic furosemide therapy ≥1 month pre-admission LVEF ≤ 40%	From admission to hospital discharge	During hospital stay	24 h urine volume and until discharge, diuretic efficiency, body weight change from admission to discharge, renal function changes, serum electrolyte changes, and dyspnea improvement during hospitalization	Dapagliflozin enhanced loop diuretic action and diuretic efficiency (34.8 ± 2.21 mL/mg loop diuretic) vs. controls (19.5 ± 1.23 mL/mg loop diuretic) No significant effect on serum potassium (4.11 ± 0.42 mEq/L) vs. controls (3.83 ± 0.50 mEq/L) or renal function [mean serum creatinine (1.39 ± 0.23 mg/dL) vs. controls (1.53 ± 0.34 mg/dL)]	In the study group, urine output, fluid loss per diuretic dose, and mean serum potassium were higher, while fluid/diuretic balance, mean total furosemide dose, furosemide/day dose, mean weight, and total daily insulin dose were significantly lower A significant increase in serum creatinine, a statistically significant reduction in serum sodium, and significant improvements in dyspnea were observed in both groups at discharge.
DAPA ACT HF-TIMI 68 [21]	SGLT2	Dapagliflozin 10 mg vs. Placebo	Age ≥18 Currently hospitalized for acute HF defined as 1. Presentation with worsening symptoms of HF 2. Objective signs or diagnostic testing consistent with volume overload 3. Intensification of acute HF therapy during admission Elevated NT-proBNP or BNP during the current hospitalization	Eligible patients randomized no earlier than 24 h and up to 14 d after presentation while still hospitalized once they have been stabilized: 1. No increase in iv diuretics in the 12 h before randomization 2. No use of intravenous vasodilators or inotropes during the 24 h before randomization	Primary endpoint window: 2 months	Primary efficacy endpoint of the trial is the time to first occurrence of cardiovascular death or a worsening HF event defined as: 1. Worsening HF during the index hospitalization requiring inotropes o mechanical/ventilatory support. 2. Re-admission to the hospital for worsening HF 3. Urgent ambulatory visit with iv diuretics	No statistically significant reduction in cardiovascular mortality or worsening HF (10.9% vs. 12.7%; (HR 0.86; 95% CI 0.68–1.08; *p* = 0.20) Higher rates of symptomatic hypotension (3.6% vs. 2.2%) and renal function decline (5.9% vs. 4.7%) without significant increase in study drug discontinuation.	Admission to randomization time 3.6 (IQR 2.1–5.4) days. Safety of dapagliflozin both in patients hospitalized for de novo or worsening HF Lack off efficacy in reducing primary endpoint probably related to short follow-up and lower-than-expected rate of events

**Table 2:** Benefits and drawbacks of SGLT2i therapy.

What We Know About SGLT2i Therapy	What We Do Not Know About SGLT2i Therapy
SGLT2i (dapagliflozin, empagliflozin) are safe to initiate in hemodynamically stable patients hospitalized for acute HF, irrespective of LVEF or diabetes status. Early use (within 2–5 days of admission) is associated with improved patient-reported outcomes and fewer HF events at 90 days (EMPULSE, SOLOIST-WHF). SGLT2i demonstrate a modest but consistent improvement in decongestion indices and loop diuretic efficiency, with minimal effects on blood pressure and renal function. Their mechanism of benefit likely extends beyond diuresis, involving metabolic and anti-oxidative myocardial effects. Continuation of pre-existing SGLT2i therapy during hospitalization appears safe in stable patients.	Whether SGLT2i initiation during the very early decongestion phase confers additional benefit compared with initiation at discharge. The extent to which their modest decongestive effect translates into improved short-term hemodynamics or renal protection. The efficacy of SGLT2i in specific high-risk subgroups (e.g., cardiogenic shock, severe renal dysfunction, advanced hypotension). Long-term prognostic impact when started during hospitalization, given the limited follow-up of existing trials (≤90 days). Their role in patients with prior heart or kidney transplantation, for whom no solid evidence currently exists.
